# All-trans retinoic acid in hematologic disorders: not just acute promyelocytic leukemia

**DOI:** 10.3389/fphar.2024.1404092

**Published:** 2024-07-04

**Authors:** Yan Chen, Xia Tong, Rongyuan Lu, Zhengfu Zhang, Tao Ma

**Affiliations:** ^1^ Department of Hematology, The Affiliated Hospital of Southwest Medical University, Luzhou, China; ^2^ Department of Hematology, Yanyuan People’s Hospital, Liangshan, China

**Keywords:** all-trans retinoic acid, differentiation, acute promyelocytic leukemia, immune thrombocytopenia, CD38, clinical trials

## Abstract

All-trans retinoic acid (ATRA) plays a role in tissue development, neural function, reproduction, vision, cell growth and differentiation, tumor immunity, and apoptosis. ATRA can act by inducing autophagic signaling, angiogenesis, cell differentiation, apoptosis, and immune function. In the blood system ATRA was first used with great success in acute promyelocytic leukemia (APL), where ATRA differentiated leukemia cells into mature granulocytes. ATRA can play a role not only in APL, but may also play a role in other hematologic diseases such as immune thrombocytopenia (ITP), myelodysplastic syndromes (MDS), non-APL acute myeloid leukemia (AML), aplastic anemia (AA), multiple myeloma (MM), etc., especially by regulating mesenchymal stem cells and regulatory T cells for the treatment of ITP. ATRA can also increase the expression of CD38 expressed by tumor cells, thus improving the efficacy of daratumumab and CD38-CART. In this review, we focus on the mechanism of action of ATRA, its role in various hematologic diseases, drug combinations, and ongoing clinical trials.

## 1 Introduction

Retinoids are a group of vitamin A metabolites that include retinal, β-carotene, retinol, isotretinoin, all-trans retinoic acid (ATRA), 9-cis retinoic acid, and 13-cis retinoic acid (Ni et al., 2019; Szymański et al., 2020). ATRA plays a role in tissue development, neurological function, immune function, reproduction, vision, cell growth and differentiation, tumor immunity, apoptosis (Niapour et al., 2015; Busada and Geyer, 2016; Hao et al., 2021; Isla-Magrané et al., 2022). Mammals cannot synthesize vitamin A on their own and get it mainly from fruits, vegetables, and animal food sources (eggs and liver). Vitamin A is hydrolyzed to retinol by retinyl ester hydrolase (REH) in the intestines, and retinol is oxidized to all-trans retinaldehyde by retinol dehydrogenase in extrahepatic cells, which is then converted to ATRA by retinaldehyde dehydrogenase (RDH) (Kedishvili, 2013; Ni et al., 2019; Liang et al., 2021). Vitamin A and its synthetic analogs regulate epidermal keratinization, differentiation, maturation, and proliferation. Retinoids are widely used in cutaneous oncology for therapeutic and chemo-prevention (non-melanoma skin cancer, primary cutaneous T-cell lymphoma) and for the treatment of inflammatory skin disorders (acne vulgaris, rosacea, melasma) and hyperproliferative disorders (ichtyosis, psoriasis) (Cosio et al., 2021). Retinoids are also used in the treatment of photodamaged skin, where primarily ATRA ameliorates UV-induced skin damage (Cosio et al., 2021). Subsequently, ATRA has also been used in the treatment of central nervous system disorders (depression), allergic disorders (asthma), metabolic disorders (atherosclerosis), infectious disorders (invasive pulmonary aspergillosis and bacterial infections), tumor immunity and other diseases (Sakamoto et al., 2015; Hu et al., 2020; Campione et al., 2021; Kalisz et al., 2021; Ma et al., 2022). In solid tumors, ATRA promotes programmed death ligand-1 (PD-L1) expression to modulate immunosurveillance in gastric cancer (GC), ATRA treats breast cancer by reversing the mesenchymal transcription programs, and disruption of ATRA signaling plays a role in driving aberrant behavior in cancer stem cells (Bobal et al., 2021; Ma et al., 2022; Brown, 2023). ATRA is not new to hematologists. It is used in the treatment of acute promyelocytic leukemia (APL) and was the first cell differentiation agent to be used for clinical approval (Wang and Chen, 2000). It is well known that APL used to be an acute myeloid leukemia (AML) with a great risk of bleeding and a high mortality rate, and that APL is caused by a balanced translocation t (15; 17) (q22; q12-21), that leads to a fusion of the promyelocytic leukemia (PML) gene with the retinoic acid receptor alpha (RARα) gene, and that the PML-RARα fusion oncoprotein induces leukemia by blocking normal myeloid cell differentiation (Yilmaz et al., 2021). The combination of ATRA and arsenic trioxide has changed the treatment strategy for APL, making it an acute leukemia that can be cured without chemotherapy regimens (Wang HY. et al., 2022; Kutny et al., 2022).

**FIGURE 1:**
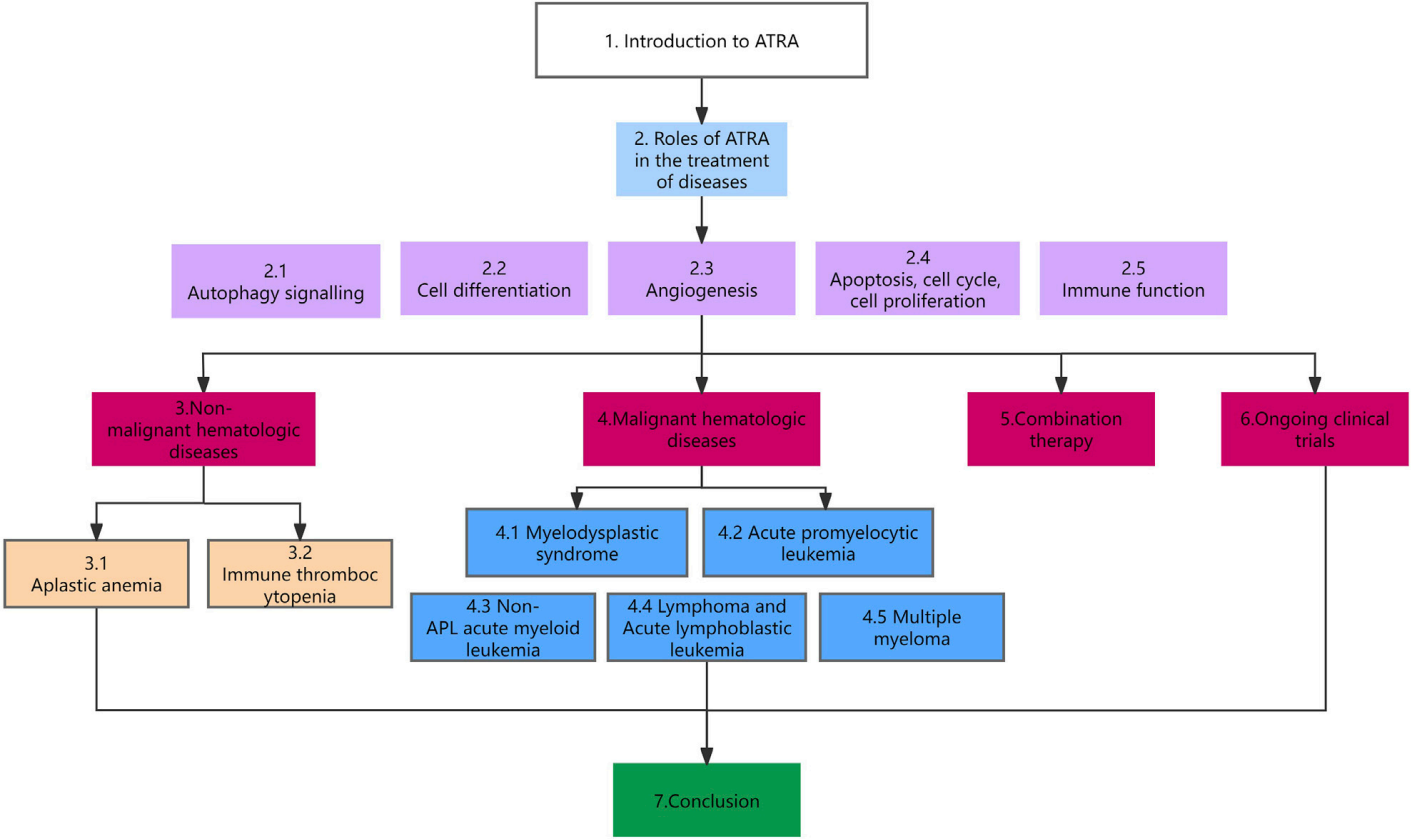
Schematic organization of this paper.

As the study of ATRA has intensified, researchers have found it to be useful in the treatment of myelodysplastic syndromes (MDS), Immune thrombocytopenia (ITP), aplastic anemia (AA), non-APL AML, and acute lymphoblastic leukemia (ALL), and it has been used as a new second-line regimen in a number of diseases. This article provides an overview of the role and mechanism of action of ATRA in hematologic diseases. The organization of this paper is shown in Figure 1.

## 2 Roles of ATRA in the treatment of diseases

### 2.1 Autophagy signalling

Autophagy is a critical homeostatic pathway that promotes degradation and recycling of cellular material (Klionsky et al., 2021). Currently, it is widely accepted that lack of autophagy promotes early tumor development due to its tumor suppressor role in normal cells (Debnath et al., 2023). Autophagy can also contribute to the development of some tumors in some cases. Rat sarcoma (RAS) activation promotes tumor proliferation and drives tumor development, but this can lead to an increase in the tumor’s demand for cellular energy metabolism and anabolic precursors. Autophagy promotes tumor development by alleviating the limited supply of external nutrients through self-digestion (Debnath et al., 2023). The PML-RARα gene inhibits PU-1-dependent transcriptional activation of several genes, including autophagy-related genes, in APL cells (Brigger et al., 2014; Jin et al., 2018). APL cells have low levels of autophagy gene expression and reduced autophagic activity. ATRA induces autophagy via the mammalian target of rapamycin pathway (mTOR), and autophagic degradation is significant for both basal turnover and therapy-induced proteolysis of PML/RARα (Isakson et al., 2010; Moosavi and Djavaheri-Mergny, 2019). The combination of ATRA and valproic acid (VPA) induces the development of autophagy in ATRA-resistant AML (Benjamin et al., 2022). ATRA-mediated crosstalk between hepatic stellate cells and Kupffer cells promotes the NACHT, LRR, and PYD domains-containing protein 3 (NLRP3) activation, inhibits autophagy, and aggravates acute liver injury (Yu et al., 2022). In breast cancer cells, ATRA induces the onset of autophagy mediated by RARα (Brigger et al., 2015). ATRA-mediated upregulation of miR-30a sensitizes GC cells to cisplatin by inhibiting autophagy (Abbasi et al., 2022). In bacterial infections, ATRA also promotes autophagy, thereby reducing the bacterial burden in human macrophages (Coleman et al., 2018). ATRA works by promoting autophagy in a variety of diseases, including acute leukemia, breast cancer, and bacterial infections.

### 2.2 Cell differentiation

PML-RARα fusion gene is the major genetic alteration in APL. PML acts as a tumor suppressor protein that accumulates in the nucleosome to induce apoptosis, whereas RARα is mainly involved in cell differentiation and renewal, and the formation of the PML-RARα fusion gene impairs the functions of PML and RARα, leading to tumor formation (di Masi et al., 2015). Physiological concentrations of ATRA (up to 90 nmol/L) bound to RARα failed to release the co-repressor of the PML-RARα multimer, and differentiated genes could not be transcribed (Borges et al., 2021). However, binding of pharmacological concentration of ATRA (around 1umol/L) to RARα, RARα transcription restarted to proceed normally, PML-RARα protein was degraded, and APL cells differentiated into polymorphonuclear granulocytes through the RAF-1/MEK/ERk signaling pathway, which reversed the malignant phenotype of APL, and the reduction in the concentration of PML protein restored apoptosis of APL cells (Nervi et al., 1998; Borges et al., 2021). Blocking RAF-1 activation reduces MEK/ERk activation and ATRA-induced cell differentiation, and ATRA plays a role in inducing APL cell differentiation by increasing the protein levels of C/EBPβ, C/EBPε, and PU.1 through the MEK/ERk pathway (Weng et al., 2016). In isocitrate dehydrogenase-2 (IDH2) mutated AML, ATRA in combination with the IDH2 inhibitor enasidenib induces cell differentiation, an effect associated with the RAF-1/MEK/ERK pathway and the induction of autophagy. Enasidenib in combination with ATRA may further improve or increase the response rate in patients with IDH2-mutated AML (Kim et al., 2020). Dasatinib in combination with ATRA could exert a synergistic effect in ATRA-resistant APL cells, and the combination of the two drugs activated the RAF-1/MEK/ERK pathway, and augmented the upregulation of ATRA-promoted PU.1, C/EBPβ, and C/EBPε protein levels, which facilitated cell differentiation (Ding et al., 2018). ATRA plays a major role in inducing cell differentiation through the RAF-1/MEK/ERK signaling pathway and activation of PU.1, C/EBPβ, and C/EBPε proteins, and the addition of dasatinib may play a synergistic role when ATRA is resistant.

### 2.3 Angiogenesis

*In vitro* experiments revealed a decrease in the expression of both vascular endothelial growth factor (VEGF) and VEGFR-2 when ATRA and melatonin were co-treated with human umbilical vein endothelial cells (HUVEC) and NB4 (APL cell line), which suggests that the combination of ATRA and melatonin has an anti-angiogenic effect (Amirzargar et al., 2023). In breast cancer, myeloid-derived suppressor cells (MDSC) are critical for resistance to antiangiogenic therapies, and *in vivo* experiments have found that ATRA treatment reduces MDSC and improves the efficacy of anti-vascular endothelial growth factor receptor 2 antibodies alone or in combination with chemotherapy (Bauer et al., 2018). In gliomas, *in vitro* experiments revealed a significant increase in the expression of VEGF and hypoxia-inducible factor-1α (HIF-1α) was observed in the low-concentration group ATRA (5 and 10 μmol/L) and a significant decrease in the expression of VEGF and HIF-1α was observed in the high-concentration ATRA group (40 μmol/L), and *in vivo* experiments revealed that high concentration of ATRA reduced microvessel density (MVD) in gliomas compared to the control group (Liang et al., 2014). *In vitro* experiments in esophageal squamous cell carcinoma, ATRA inhibited angiogenesis by decreasing the protein levels of angiopoietin-1, angiopoietin-2, and the receptor Tie-2 (Li et al., 2017). In hepatocellular carcinoma, MDSC are key drivers in maintaining an immunosuppressive tumor microenvironment, and *in vivo* experiments have revealed that ATRA induces differentiation of MDSC into mature myeloid cells, inhibits angiogenic markers, and ATRA moves towards an anti-tumor phenotype by altering the relative ratio between pro- and anti-tumor immune cells (Li X. et al., 2023). Vessel formation and *in vivo* wound healing were significantly enhanced in ATRA-treated mesenchymal stem cells (MSCs) (Pourjafar et al., 2017). Therapeutic doses of ATRA inhibit angiogenesis most of the time, but may promote angiogenesis at low concentrations.

### 2.4 Apoptosis, cell cycle, cell proliferation

ATRA increases the apoptotic effect of the apoptosis inducer ABT-737 (a bcl-2 selective inhibitor) on AML (Buschner et al., 2021). ATRA treatment of myeloid leukemia cell line K562 resulted in a significant increase in apoptosis, cell cycle arrest in the G0/G1 phase, and a decrease in cell proliferation, which was achieved by up-regulating the increased expression of homeobox A5 (HOXA5) (Liu et al., 2016). ATRA induced cell cycle arrest, increased apoptosis, and decreased cell proliferation in APL cell line NB4, and this effect was exerted by inducing retinoic acid-inducible gene-I (RIG-I) expression and further through the AKT-FOXO3A signaling pathway (Chen et al., 2017). ATRA treatment induces a persistent inhibition of B cell lymphoma-2 (BCL-2) in non-APL AML cells, first upregulating and then decreasing myeloid cell leukemia 1 (MCL-1). Activation of the MEK/ERK and PI3K/Akt pathways by ATRA leads to activation of p90 ribosomal S6 kinase (90RSK) and inactivation of glycogen synthase kinase 3 beta (GSK3β), which increases MCL-1 levels. Sorafenib reverses the activation of p90RSK and inactivation of GSK3β, thereby blocking the increase in MCL-1 and maintaining the sustained inhibition of BCL-2, ultimately increasing apoptosis in non-APL AML (Wang et al., 2016). ATRA can also induce increased apoptosis in glioma cells *in vitro* (Wu et al., 2020). The combination of ATRA with other drugs to increase apoptosis is a future research direction.

### 2.5 Immune function

As we have mentioned before, ATRA in hepatocellular carcinoma reduces MDSC infiltrated in the tumor, increases the infiltration of cytotoxic T-cells, and moves the tumor microenvironment toward an anti-tumor phenotype (Li X. et al., 2023). ATRA enhances the expression of B-cell maturation antigen (BCMA) on MM cells, thereby improving the efficacy of chimeric antigen receptor (CAR) T-cell therapy (García-Guerrero et al., 2023). Also, ATRA upregulates the expression of CD38 in MM cells, and this upregulation is dependent on the t (4; 14) translocation. t (4; 14) translocation-induced nuclear receptor-binding SET domain-containing 2 (NSD2) is positively correlated with the ATRA-induced CD38 expression level, and ATRA enhances the efficacy of anti-CD38 CAR T cells against NSD2-high MM cells (Peng et al., 2023). Guarrera L et al. (Guarrera et al., 2023) performed RNA sequence studies on 13 GC cell lines, for which ATRA had an antiproliferative effect, and constructed a model consisting of 42 genes whose expression was correlated with ATRA sensitivity, and they used these data to analyze GC RNA sequences in the in the Cancer Genome Atlas/Cancer Cell Line Encyclopedia (TCGA/CCLE) database. They found that 45% of TCGA GCs were sensitive to ATRA, which had a significant immunomodulatory effect that was controlled by upregulation of interferon regulatory factor 1 (IRF1). ATRA and immune checkpoint inhibitors are a reasonable combination for the treatment of GC. However, a study by Ma ZL et al. (Ma et al., 2022) found that ATRA antagonized the function of PD-L1 antibody in GC, and ATRA induced the expression of PD-L1 in GC, which made GC cells highly resistant to killing by activated T cells. ATRA can greatly weaken the immunosuppressive effects of infiltrating MDSC and also activate the antitumor effects of CD8^+^ T cells, and the combination of ATRA with immune checkpoint inhibitors, tumor vaccines, and chemotherapy has been widely studied in a variety of tumors (Bi et al., 2023). ATRA not only plays a crucial role in inducing intestinal tropism of lymphocytes and regulating T helper cell differentiation, but is also an important regulator of innate immune cells such as tolerogenic dendritic cells (DCs) and innate lymphoid cells (ILCs) (Czarnewski et al., 2017). The poor aqueous solubility and rapid metabolism of ATRA have limited its application as an immunomodulator for anticancer immunotherapy. Liposomal ATRA (L-ATRA) is a drug with release persistence, and actively loaded L-ATRA achieves stable encapsulation and controlled release and accumulation of the drug in tumor tissues; they promote the maturation of MDSCs into DCs and the immune response against cancer cells. L-ATRA can prevent tumor growth when used as monotherapy, and its anticancer activity is higher when L-ATRA is combined with checkpoint inhibitors (Zheng et al., 2022). The modulation of tumor immunity by ATRA and the combination of ATRA with immunosuppressive agents deserve more attention.

**FIGURE 2:**
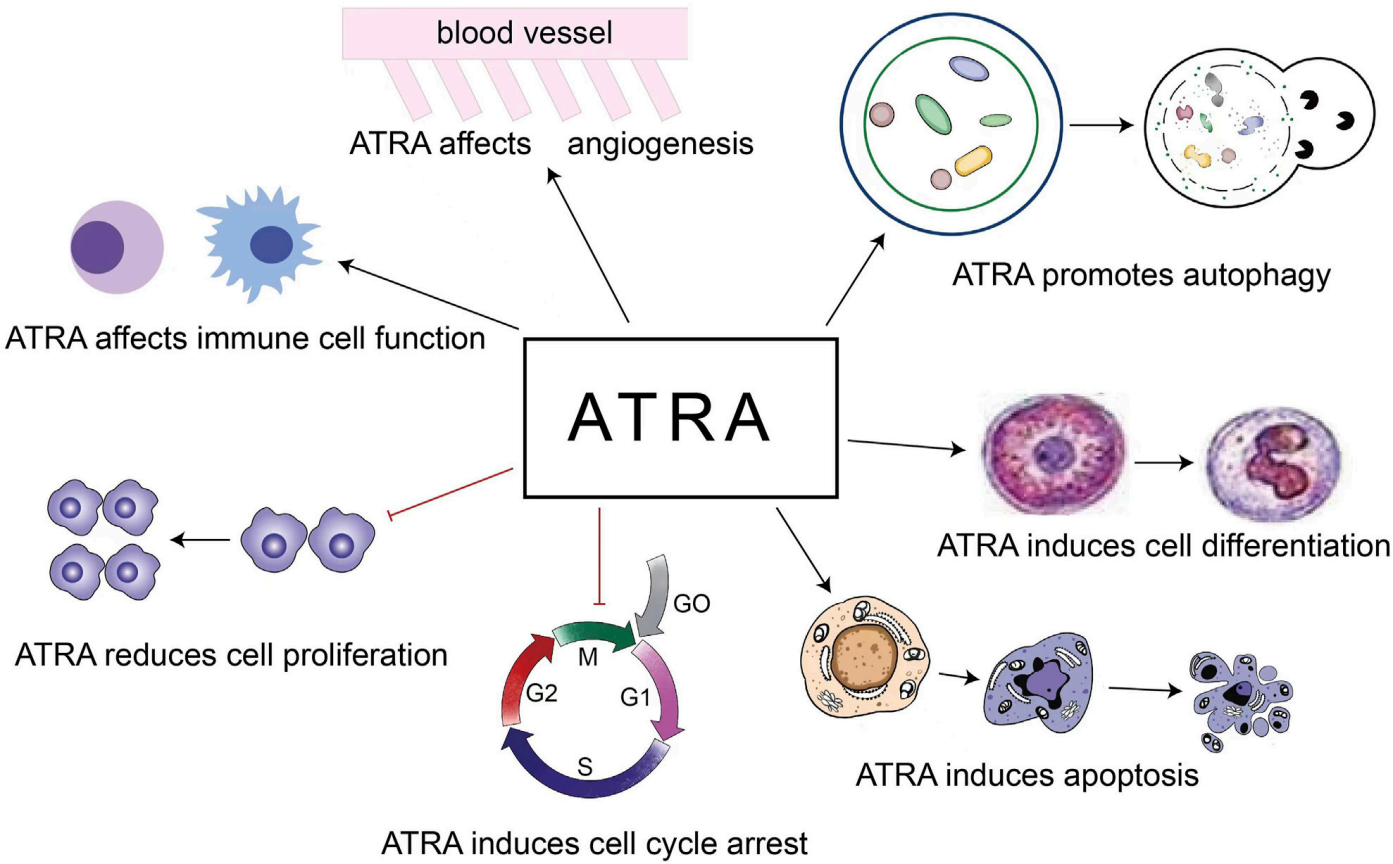
Roles of ATRA in the treatment of diseases.

The role of ATRA in the treatment of diseases is based on the above 5 points (Figure 2). Our review focuses on the role of ATRA in the treatment of hematologic diseases, especially the new applications in the last 5 years, and first we look at the role of ATRA in non-malignant hematologic diseases.

## 3 Non-malignant hematologic diseases

### 3.1 Aplastic anemia

Aplastic anemia (AA) is a bone marrow failure syndrome that manifests itself as a decrease in total blood count. AA is an immune-mediated disease caused primarily by abnormally active T-cell function that attacks hematopoietic stem and progenitor cells. Symptoms in most patients with AA improve with the use of anti-thymocyte globulin and cyclosporine to inhibit T-cell function (Young, 2018). The balance between T helper (Th) 1 cells and Th2 cells is important for a normal immune response, and the two work together to maintain immune homeostasis. AA is characterized by an overreaction of Th1 cells and overproduction of interferon-γ (IFN-γ), and a decrease in regulatory T cells (Tregs) (Tang et al., 2020). ATRA maintains the balance between T cell subsets and exhibits strong immunomodulatory capacity (Brown et al., 2015; Erkelens and Mebius, 2017). In the model of AA treated with ATRA, ATRA inhibited the proliferation, activation and effector functions of T cells and inhibited the Fas/FasL pathway, caused Th1 cells to develop into Th2 cells by targeting the nuclear factor of activated T cells (NFAT) pathway, promoted Th2 cells by upregulating JunB, and ultimately rebalance T cell subsets (Tang et al., 2020). ATRA also inhibits the differentiation of Th17 cells, promotes the development of Tregs, and reduces abnormally activated T cells in the bone marrow microenvironment. ATRA ameliorates bome marrow (BM) dysplasia and pancytopenia in AA mice, and ATRA is a potent agent for improving the therapeutic outcome of AA (Tang et al., 2020).

### 3.2 Immune thrombocytopenia

Immune thrombocytopenia (ITP) is an autoimmune disease in which DCs play a crucial role in the destruction of self-tolerance, and MSCs promote the development of regulatory DCs (regDCs) (Xu et al., 2017). MSCs become senescent and apoptotic in ITP, with a reduced immunosuppressive effect on T cells and B cells. The impaired ability of MSCs in ITP patients to induce CD34+-regDCs is associated with the Notch-1/Jagged-1 signaling pathway. ATRA partially corrects the impairment of MSCs in ITP patients and partially restores the ability of MSCs in ITP patients and healthy controls to induce CD34+-derived regDCs, which is therapeutic for ITP (Xu et al., 2017). The PI3K/AKT signaling pathway is involved in the process of MSCs deficiency. MiR-98-5p can act by targeting insulin-like growth factor 2 mRNA-binding protein 1 (IGF2BP1), and the subsequent downregulation of insulin-like growth factor 2 (IGF-2) inhibits the PI3K/AKT signaling pathway, and miR-98-5p can also upregulate the expression of p53. ATRA could protect MSCs and reduce apoptosis of MSCs by downregulating miR-98-5p (Wang et al., 2020). Macrophages differentiate into either a pro-inflammatory M1 phenotype or an anti-inflammatory M2 phenotype in response to different environmental stimuli. ITP patients have more polarized M1 macrophages in the spleen and impaired immune function. High-dose dexamethasone or ATRA can correct this imbalance by decreasing the expression of M1 markers and increasing the expression of M2 markers, and ATRA modulation of macrophages can shift the T-cell cytokine profile toward Th2 (Feng Q. et al., 2017).

The previous paragraph mainly describes the mechanism of action of ATRA in the treatment of ITP, and below we look at the data from the relevant clinical trials. Dai L et al. (Dai et al., 2016) treated 35 patients with relapsed refractory ITP with ATRA (10 mg tid). Complete and overall responses were observed in 10 (28.6%) and 19 (54.3%) patients, and, treatment with ATRA significantly increased the proportion of Treg cells, IL-10 levels, and forkhead box P3 (Foxp3) expression (all three of which were significantly decreased in ITP patients). The study by Feng FE et al. (Feng FE. et al., 2017) randomly assigned ITP patients in a 1:1 ratio to receive either oral ATRA (10 mg bid) + oral danazol (200 mg bid) or oral danazol monotherapy (200 mg bid) for 16 weeks 45 patients were enrolled in the ATRA + danazol group, and 48 in the danazol group. 28 patients (62%) treated with ATRA + danazol achieved a sustained response compared with 12 patients (25%) in the danazol monotherapy group, a statistically significant difference between the two groups. Wu YJ et al. (Wu et al., 2022) randomized ITP patients in a 2:1 ratio, with 112 patients receiving low-dose rituximab (LD-RTX) (100 mg qw for a total of 6 weeks) + ATRA (20 mg/m^2^ for 12 weeks), and 56 receiving LD-RTX monotherapy; there was an overall response of 80% in the LD-RTX + ATRA group compared to 59% in the LD-RTX 59% in the monotherapy group. ATRA significantly improves initial and long-term responses in patients with ITP and is associated with reduced rates of relapse and salvage therapy, with the main adverse effects being dry skin, dizziness, headache, and acne-like rashes (Yang et al., 2023). ATRA has provided a new boon in the treatment of ITP patients.

## 4 Malignant hematologic diseases

### 4.1 Myelodysplastic syndrome

Myelodysplastic syndrome (MDS) is a heterogeneous disorder characterized by hypoplasia, disturbed differentiation of hematopoietic cells, and increased apoptosis of diseased cells leading to a decrease in one or more lineages, and patients with high-risk MDS (HR-MDS) are predisposed to progression to AML (Trivedi et al., 2021). Decitabine (DAC) is one of the main drugs used in the treatment of HR-MDS, and DAC alone may be resistant by a mechanism that induces NF-E2-related factor 2 (Nrf2) activation and a downstream antioxidant response that inhibits reactive oxygen species (ROS) generation, leading to drug resistance. The combination of DAC + ATRA decreased the viability of MDS cells, delayed the progression of tumor cells, and prolonged the survival time of mice, and ATRA increased the efficacy of DAC by blocking Nrf2 activation through activation of the RARα-Nrf2 complex, which led to ROS accumulation and ROS-dependent cytotoxicity (Wang et al., 2023). USO1 is a vesicular transporter involved in endoplasmic reticulum to Golgi transport of proteins. USO1 is aberrantly activated in MDS *in vivo* and *in vitro*, and silencing of USO1 promotes myeloid differentiation and inhibits proliferation of MDS cells. USO1 exerts its oncogenic effects by inactivating Raf/ERK signaling (Li et al., 2021). 4-Amino-2-Trifluoromethyl-Phenyl Retinate (ATPR) is a ATRA derivative, and ATPR can induce MDS remission *in vitro* by decreasing USO1 and modifying Raf/ERK signaling (Li et al., 2021). ATPR also increases apoptosis in MDS cells via p53, and this apoptosis is interdependent with activation of the BNIP3 gene (Du et al., 2018). ATRA or ATPR has a pro-apoptotic effect on MDS cells and ATRA overcomes DAC resistance, next we look at the results of clinical trials of ATRA for MDS.

Low-dose cytarabine or hydroxyurea in combination with or without ATRA in elderly patients with MDS, low-dose cytarabine improved the remission rate of MDS, but there was no better efficacy in combination with ATRA (Burnett et al., 2007). VPA monotherapy for MDS resulted in a response in 44% (8) of patients, including 1 partial remission, and 4 of 5 relapsed patients were treated with VPA + ATRA, with 2 responding again; VPA + ATRA for MDS may be efficacious (Kuendgen et al., 2004). VPA + ATRA treatment of low-risk MDS (LR-MDS) patients resulted in significant freedom from platelet transfusions lasting several months in approximately 30% of patients, reducing the burden of palliative care and improving quality of life (Pilatrino et al., 2005). VPA + ATRA treatment of patients with MDS resulted in improvement in hematologic status in only 18 (24%) of 75, with a median duration of response of 4 months (Kuendgen et al., 2005). Addition of demethylating drugs improves the response rate of VPA + ATRA for MDS. Soriano AO’s study of AML or HR-MDS treated with azacitidine (AZA) + VPA + ATRA had an overall response rate of 42% in 53 patients, 52% in previously untreated elderly patients, and a median duration of remission of 26 weeks (Soriano et al., 2007). Zhang W et al. (Zhang et al., 2006)treated 63 patients with primary refractory anemia (RA) with ATRA + androgens + calcitriol, and 68.3% (43) of the patients showed an improvement in their hematological status, with overall survival rates of 68.72% and 53.18% at 3 and 5 years, respectively. In 31 patients (13 AML, 18 MDS) with myeloid tumors unsuitable for intense chemotherapy treated with low-dose DAC + ATRA, complete remission was achieved in 7 patients (22.6%) and partial remission in 4 patients (12.9%), resulting in an overall remission rate of 58.1%, a median overall survival of 11.0 months, and a 1-year OS rate of 41.9% and a 2-year OS rate of 26.6% (Wu et al., 2016). ATRA + erythropoietin (EPO) treated 27 patients with low- or intermediate-risk MDS; 13 patients (48%) experienced a clinically significant erythrocyte response with a rise in hemoglobin level of at least 1 g/dL or a reduction in transfusion requirements, 5 of 12 neutropenic patients had a neutrophilic response, 6 of 9 thrombocytopenic patients had platelet responses, and three patients had a whole blood cell response; all patients had tolerable side effects (Stasi et al., 2002). Another clinical trial found that EPO + ATRA could be used to treat patients with MDS anemia in whom erythropoiesis-stimulating agents (ESAs) alone were ineffective, but not in patients with high levels of endogenous EPO, and it did not improve neutropenia or thrombocytopenia (Itzykson et al., 2009).

Clinical trials of ATRA for the treatment of MDS were carried out in large numbers 20 years ago, including ATRA combined with VPA, EPO, androgens, cytarabine, and hydroxyurea, etc. The efficacy of ATRA combined with EPO or androgens in the treatment of RA is worthy of affirmation, but the treatment of HR-MDS did not obtain too good clinical efficacy, and the subsequent application in the clinic is also less. However, ATRA overcoming the resistance of DAC opens a new window for ATRA treatment of MDS, and the efficacy of ATRA combined with demethylating drugs in the treatment of MDS deserves further study.

### 4.2 Acute promyelocytic leukemia

APL accounts for approximately 10%–15% of all AML and is characterized by leukocyte abnormalities, anemia, low platelets, coagulation abnormalities, and severe bleeding, with a major chromosomal abnormality of t (15; 17) (q22; q12-21), which produces the PML-RARα fusion gene (Yilmaz et al., 2021). In the 1980s, ATRA was used to treat APL, and all 24 patients achieved complete remission, but the duration of complete remission with ATRA monotherapy for APL was short (Huang et al., 1988). After the status of ATRα monotherapy in APL treatment was established, ATRA in combination with chemotherapy for APL became the mainstream regimen, and ATRA in combination with chemotherapy reduces the relapse rate of patients, and patients on maintenance therapy with ATRA achieve significantly better survival (Fenaux et al., 1999). Arsenic trioxide (ATO, As2O3), a chemical derived from the ancient Chinese poison arsenic, was identified in the 1990s for the treatment of APL. High concentrations of ATO induced apoptosis in APL cells, while low concentrations induced differentiation, and ATO induced apoptosis in NB4 cells through the downregulation of Bcl-2 expression and regulation of the PML-RAR alpha/PML protein (Chen et al., 1996; Zhao et al., 2001). A clinical trial divided APL patients into ATRA + ATO *versus* ATRA + chemotherapy groups; ATRA + ATO enrolled 77 patients, all of whom achieved complete remission, and ATRA + ATO enrolled 79, 95% of whom achieved complete remission. With a median follow-up of 34.4 months, the 2-year event-free survival rate was 97% in the ATRA + ATO group and 86% in the ATRA + chemotherapy group. ATRA + ATO was associated with less hematologic toxicity and fewer infections than ATRA + chemotherapy (Lo-Coco et al., 2013). ATRA + ATO is a viable treatment with a high cure rate, low recurrence rate, no difference in survival, and a low incidence of liver toxicity compared to ATRA + idarubicin (Burnett et al., 2015). Kutny MA used an ATRA + ATO-based regimen to treat pediatric APL patients. The 2-year event-free survivalrate was 98.0% and the overall survival rate was 99.0% in patients with standard-risk APL, and 96.4% and 100% in patients with high-risk APL; the regimen was associated with a shorter duration of treatment, fewer days of hospitalization, and less exposure to anthracyclines and intrathecal chemotherapy (Kutny et al., 2022). Abaza Y et al. (Abaza et al., 2017) treated APL with ATRA + ATO and combined gemtuzumab ozogamicin (GO) in high-risk patients and in low-risk patients who developed leukocytosis during induction. A total of 187 patients were treated, including 54 high-risk and 133 low-risk patients. The complete remission rate was 96%, the mortality rate on induction therapy was 4%, and there were only 7 relapses. There is no doubt that ATRA + ATO for APL is one of the most successful protocols studied in recent years, bringing an AL with severe bleeding and high mortality to a curable state.

### 4.3 Non-APL acute myeloid leukemia

ATRA has significantly improved the prognosis of APL patients, and its study in non-APL AML is so extensive that scientists are eager to find new breakthroughs. But ATRA alone does not work well, and more research is focusing on combinations. An inhibitor of MEK, trametinib, in combination with ATRA restored the sensitivity of ATRA-resistant AML cell lines to ATRA by enhancing the protein levels of STAT3 and phosphorylation of Akt or JNK, and STAT3, PI3K, and JNK inhibitors all inhibited trametinib + ATRA induced cell differentiation in AML cell lines (Lu et al., 2021). ATRA inhibits translation and protein synthesis in AML cells, and the genes regulated by ATRA are mainly concentrated in the PI3K/AKT signaling pathway, and ATRA + PI3K/AKT inhibitor induces a large number of apoptotic cells and greatly inhibits cloning of AML cells (Wang K. et al., 2022). ATRA treatment of differentiation-responsive AML cells induces persistent inhibition of BCL-2, while first upregulating and then decreasing the level of MCL-1. ATRA activates p90RSK and inactivates GSK3β, and increases the translation and stability of MCL-1 to raise its level. sorafenib reverses the activation of p90RSK and the inactivation of GSK3β, blocks the increase of MCL-1, and maintains the decrease of BCL-2 level, thus enhancing apoptosis in non-APL AML cell lines (Wang et al., 2016). Low dose midostaurin (0.25–0.5 μM) + ATRA induced cell differentiation, whereas high dose midostaurin (0.25–0.5 μM) + ATRA led to apoptosis in FMS-like tyrosine kinase-3 (FLT3) AML cell line, and apoptosis induced by high dose midostaurin + ATRA was dependent on caspase-3/7. Low dose midostaurin + ATRA inhibited the activation of Akt, leading to dephosphorylation of RAF S259, activation of RAF/MEK/ERK, and upregulation of the protein levels of C/EBPβ, C/EBPε, and PU.1, which resulted in an increase in cell differentiation (Lu et al., 2022). Nucleophosmin-1 (NPM1) mutations in AML not only dislocate NPM1 from the nucleolus but also disorganize the PML nucleosome, ATRA in combination with ATO synergistically induces proteasomal degradation of NPM1 mutations in AML cell lines or primary samples leading to differentiation and apoptosis, and downregulation of the NPM1 mutant by ATRA + ATO also enhances the therapeutic response of AML cells to doxorubicin (El Hajj et al., 2015; Martelli et al., 2015). Targeted ferroptosis contribute to ATPR-induced AML cell differentiation, ATPR induces ferroptosis in a dose-dependent manner, and ATPR-induced ferroptosis is regulated by autophagy through iron homeostasis, especially Nrf2 (Du et al., 2020). Both cyclin-dependent kinase 2 (CDK2) depletion and CDK2 pharmacological inhibitors significantly sensitize AML cell lines to ATRA-induced cell differentiation (Shao et al., 2020). It has also been found that HIF2α blockade in cooperation with ATRA triggers AML cell differentiation (Magliulo et al., 2023). One of the causes of resistance to ATRA in non-APL AML is the aberrant acetylation of the acetyltransferase general control non-depressible 5 (GCN5) via histone 3 lysine 9 (H3K9ac) residues, and GCN5 also maintains the expression of stemness and leukemia-associated genes (Kahl et al., 2019). Inhibition of GCN5 increases the therapeutic response to ATRA, and concomitant inhibition of the lysine demethylase 1 (LSD1) enhances this response, leading to differentiation of most non-APL AMLs (Kahl et al., 2019). ATRA in combination with tranylcypromine (TCP), which inhibits LSD1, induces AML cell differentiation. A phase I/II clinical trial studied TCP/ATRA for relapsed/refractory AML with an overall response rate of 20%, median survival of 3.3 months, and 1-year survival of 22% in 18 patients, and they observed elevated levels of lysine 4 of histone H3 (H3K4me1) and lysine 4 residue on histone 3 (H3k4me2) in some of the treated patients (Wass et al., 2021). A meta-analysis found that chemotherapy + ATRA did not improve patients’ overall survival compared to chemotherapy alone (Küley-Bagheri et al., 2018). AML patients with adverse genetic features such as complex, monosomal karyotypes and TP53 lesions have a very poor prognosis, and DAC + ATRA has a synergistic antileukemic effect, increasing response rates and prolonging overall survival (Meier et al., 2022). CAR-T therapies are currently less effective in AML, but ATRA increases the expression of CD38 on the cell surface of AML cells, thereby increasing the efficacy of CD38-CAR-T for AML (Yoshida et al., 2016). The use of ATRA as maintenance therapy for AML in children has comparable efficacy to the use of cytarabine (Li J. et al., 2023).

The basic research and clinical trials of ATRA in the treatment of non-APL AML are relatively numerous, and the basic research is mainly oriented to the combination of drugs, which can play a synergistic role in inducing AML cell apoptosis and cell differentiation. The efficacy of previous clinical trials has not been too encouraging, but the efficacy of the combination of new drugs (e.g., CDK2 inhibitors, daratumumab, midostaurin, etc.) and ATRA in the treatment of non-APL AML still deserves to be further investigated.

### 4.4 Lymphoma and acute lymphoblastic leukemia

CD38-CART inhibits the growth of CD38-overexpressing mantle cell lymphoma (MCL), Waldenstrom’s macroglobulinemia (WM), NK/T-cell lymphoma (NKTCL), multiple myeloma (MM), and T-cell acute lymphoblastic leukemia (T-ALL) *in vitro*, and ATRA enhances a wide range of CD38-low expressing cancer cells’ CD38 expression and increases the therapeutic efficacy of daratumumab (CD38-targeting antibody) and CD38-CART (Wang X. et al., 2022). Primary exudative lymphoma (PEL) is a lymphoma that often expresses CD38, but despite high CD38 expression, daratumumab does not induce complement-dependent cytotoxicity (CDC) in PEL but increases antibody-dependent cell-mediated cellular cytotoxicity (ADCC), and ATRA and pomalidomide significantly increase the CD38 levels in low CD38-expressing PEL cell lines, which in turn increases daratumumab-induced ADCC, and daratumumab in combination with ATRA or pomalidomide is a potential therapeutic option for PEL (Shrestha et al., 2023). Stauffer RG used nanodisks to target drug delivery to tumor cells, and they found that the combination of curcumin-nanodisks and ATRA-nanodisks significantly enhanced the bioactivity of these drugs against MCL and follicular lymphoma cells (Stauffer et al., 2015). Many T-ALL cells express CD38 on their surface, and treatment of T-ALL cells with ATRA increases CD38 expression, which further enhances antibody-dependent cellular phagocytosis (ADCP) in macrophages and ADCC, especially when using daratumumab-IgA2 (Baumann et al., 2022). ATRA-treated Ink4-Arf deletion (Arf−/−) BCR-ABL ALL cells showed increased apoptosis, fewer S-phase cells, and more G0/G1-phase cells, and ATRA reduced BCR-ABL ALL cell viability by signaling through the retinoid X receptor (Annu et al., 2020).

The primary mechanism of action of ATRA in the treatment of lymphoid system tumors is to increase the expression of CD38 on the surface of tumor cells, thereby increasing the efficacy of daratumumab or CD38-CART.

### 4.5 Multiple myeloma

A hypersialylated tumor cell surface contributes to aberrant cell migration and drug resistance, and hypersialylation has also been associated with evasion of natural killer (NK) cell-mediated immunosurveillance (Daly et al., 2022). Desialylation with sialylase and sialyltransferase inhibitor (SIA) greatly enhanced NK cell-mediated cytotoxicity against MM cells. MM cells with low CD38 expression elicited potent NK cytotoxic responses after treatment with ATRA, SIA and daratumumab (Daly et al., 2022). ATRA enhances CD38 expression, thereby increasing the efficacy of CD38-CART (Nijhof et al., 2015; Wang X. et al., 2022; Sharma et al., 2024). However, inhibition of histone deacetylase (HDAC) 6 antagonized the upregulation of CD38 expression by IFN-α and ATRA in MM cells (Bat-Erdene et al., 2019). While ATRA-induced CD38 upregulation on MM target cells can also induce CD38 levels on CD38 wild-type NK cells, ATRA-induced CD38 upregulation in MM may be counteracted by increased NK cell fratricide and impaired NK cell function, thereby reducing the overall efficacy of daratumumab, whereas deleting the CD38 level on NK cells expanded *ex vivo* using the CRISPR/Cas9 system could mitigate this situation (Naeimi et al., 2020). A clinical trial investigating the efficacy of ATRA + daratumumab in daratumumab-refractory MM found an overall response rate of 5%, with the majority of patients (66%) having stable disease (Frerichs et al., 2021). ATRA temporarily increased CD38 expression on immune cell subsets, but the clinical efficacy of the combination was suboptimal, probably because ATRA also concomitantly increased CD38 expression on NK cells, whereas daratumumab killed CD38-positive NK cells, reducing the daratumumab-mediated ADCC effect and leading to decreased efficacy (Naeimi et al., 2020; Frerichs et al., 2021). ATRA upregulates CD38 in MM cells in a nonlinear manner, and this upregulation is dependent on the t (4; 14) translocation. Whereas t (4; 14) translocation-induced NSD2 was positively correlated with ATRA-induced CD38 expression levels, NSD2 interacted with the ATRA receptor RARα to protect it from degradation (Peng et al., 2023). Knockdown of NSD2 attenuates MM sensitivity to ATRA-induced CD38 upregulation. ATRA enhances the efficacy of CD38-CAR T cells against NSD2-high MM cells both *in vitro* and *in vivo* (Peng et al., 2023). ATRA treatment leads to an increase in BCMA transcript and protein expression in MM cell lines and primary MM cells and enhances the efficacy of BCMA-CART, and co-treatment of MM cells with ATRA and the γ-secretase inhibitor, crenigacestat, further increases BCMA expression and enhances the efficacy of BCMA-CART (García-Guerrero et al., 2023). ATRA enhanced the sensitivity of MM cells to carfilzomib-induced cytotoxicity and re-sensitized carfilzomib-resistant MM cells to carfilzomib (Wang Q. et al., 2022). Mechanistically, ATRA mainly activated the retinoic acid receptor (RAR)γ and interferon-β response pathways to upregulate the expression of IRF1, which initiated the transcription of OAS1, which synthesized 2–5A upon binding to carfilzomib-induced double-stranded RNA and led to degradation of the cellular RNA by RNase L and cell death, and the selective RARγ agonist BMS961 could also re-sensitize MM cells to carfilzomib *in vitro* (Wang Q. et al., 2022).

ATRA alone has no effect on MM cells, and the mechanism of action of ATRA in MM is mainly to increase the expression of CD38 and increase the efficacy of CD38-CART, especially in MM cells with high NSD2 expression. ATRA + daratumumab, however, did not increase efficacy in patients with daratumumab-resistant MM. ATRA also increased BCMA expression, which increased the efficacy of BCMA-CART. ATRA also enhanced the sensitivity of MM cells to carfilzomib-induced cytotoxicity and re-sensitized carfilzomib-resistant MM cells to carfilzomib.

## 5 Combination therapy

ATRA plays a very important role in the treatment of APL, but the combination with ATO can still further improve the efficacy. The treatment of ATRA in hematologic diseases is mainly based on the use of combination drugs. Here we summarize the efficacy, mechanism of action of ATRA in combination with other drugs in hematologic diseases (Table 1).

**TABLE 1:** Combination of ATRA and other drugs in hematologic disorders.

Diseases	Combination of drugs	Efficacy	Mechanisms of action	References
Immune thrombocytopenia	low-dose rituximab	Overall response rate of 80%, sustained response rate of 61%	ATRA partially corrects the impairment of MSCs	Wu et al. (2022)
	danazol	Sustained response rate of 62%	ATRA restores reduced regulatory T cell and interleukin 10 concentrations and decreases FOXP3 expression	Feng et al. (2017b)
Myelodysplastic syndrome	Decitabine	Combination therapy reduced the viability of MDS cells *in vitro* and prolonged the survival time of mice	ATRA can block Nrf2 activation by activating the RARα-Nrf2 complex, leading to ROS accumulation and ROS-dependent cytotoxicity and reducing DAC resistance	Wang et al. (2023)
	VPA	Hematologic status improved in 24% of patients with a median sustained response time of 4 months	-	Kuendgen et al. (2005)
	5-azacitidine + VPA	The overall response rate was 42%. In older patients who had not received prior treatment, the response rate was 52%	DNA methylation was significantly reduced and histone acetylation was induced	Soriano et al. (2007)
	EPO	Erythrocyte response rate of 49%	Induce erythroid differentiation	Itzykson et al. (2009)
Myeloid neoplasms	Decitabine	The overall remission rate was 58.1% and the 1-year overall survival was 41.9%	DAC synergized with ATRA on growth inhibition, differentiation and apoptosis of tumor cells, and ATRA enhanced the effect of DAC on p16 demethylation	Wu et al. (2016)
Acute promyelocytic leukemia	ATO	The 2-year event free survival rates for standard-risk and high-risk patients were 98.0% and 96.4%, respectively, and the overall survival rates were 99.0% and 100%, respectively	Induces differentiation and promotes apoptosis	Kutny et al. (2022)
	ATO + gemtuzumab ozogamicin	Complete responses were achieved in 86% of patients, with 3-year event-free and overall survival rates of 78% and 86%, respectively	Induces differentiation and promotes apoptosis, humanized anti-CD33 antibody-drug conjugate	Lancet et al. (2020)
	ATO + gefitinib	Reduced proliferation and survival of ATRA-resistant APL cells	Promoting myeloid differentiation of ATRA and ATO-resistant APL cells	de Almeida et al. (2021)
	Rosmarinic acid (RA)	Enhancement of ATRA-induced macrophage differentiation in APL cells	ATRA/RA induces the expression of CD11b	Heo et al. (2015)
	HDAC3 inhibitor + ATO	Induction of APL cell differentiation, apoptosis and reduction of cell self-renewal	Inhibition of HDAC3 promotes PML-RARα ubiquitination and degradation and reduces PML-RARα expression in wild-type and ATRA- or ATO-resistant APL cells	Dai et al. (2023)
Non APL AML	GCN5 inhibitor	Induction of non-APL AML differentiation	GCN5 maintains expression of stemness and leukemia-associated genes through aberrant acetylation of H3K9ac residues leading to resistance of non-APL AML to ATRA	Kahl et al. (2019)
	PI3K/AKT inhibitor	Induces massive apoptosis, greatly inhibits AML cell cloning, and inhibits FLT3-ITD-driven transformation of CD34^+^ hematopoietic stem/progenitor cells	PI3K/AKT promotes AML cell stemness and ATRA requires activation of PI3K/AKT to effectively induce AML cell differentiation	Wang et al. (2022b)
	Sorafenib	Enhancement of ATRA-induced apoptosis in non-APL AML cell lines and primary AML cells	Reverses p90RSK activation and GSK3β inactivation, thereby blocking ATRA-induced increase in Mcl-1 and maintaining suppressed Bcl-2 level	Wang et al. (2016)
	notopterol	Enhanced induction of differentiation	Induction of apoptosis, differentiation and G0/G1 arrest in human AML cells	Huang et al. (2019)
	Tranylcypromine (TCP)	TCP/ATRA treated 18 patients with relapsed/refractory AML with an overall response rate of 20%, a median overall survival of 3.3 months, and a 1-year survival rate of 22%	Elevated levels of H3K4me1 and H3k4me2 were observed in AML cells and leukocytes from patients. TCP/ATRA induced AML leukocyte differentiation	Wass et al. (2021)
	Midostaurin	Antitumor activity against wild-type FLT3 mutant AML *in vitro* and *in vivo*	Inhibition of Akt activation leads to dephosphorylation of RAF S259 and activation of RAF/MEK/ERK, as well as upregulation of protein levels of C/EBPβ, C/EBPε and PU.1	Lu et al. (2022)
	CDK2 inhibition	Induction of AML cell differentiation	Transcriptional activation of differentiation and maturation pathways	Shao et al. (2020)
	CD38-CAR T	CD38-specific T cells enhance cytotoxicity against AML cells via ATRA	ATRA increases CD38 expression	Yoshida et al. (2016)
Primary effusion lymphoma	daratumumab	Increased daratumumab-induced ADCC	ATRA significantly increases surface CD38 levels in low CD38-expressing PEL cell lines	Shrestha et al. (2023)
Lymphoid malignancies	CD38-CAR T	Enhanced anti-tumor activity of daratumumab and CD38-CAR T cells in xenograft tumors	ATRA increases CD38 expression in a variety of CD38 low-expressing cancer cells	Wang et al. (2022c)
T-acute lymphoblastic leukemia	Daratumumab-IgA2 isotype switch variant	Enhanced ADCP and ADCC	ATRA increases CD38 expression	Baumann et al. (2022)
Multiple myeloma	CD38-CART	ATRA enhances the efficacy of anti-CD38-CART cells against NSD2-high MM cells both *in vitro* and *in vivo*	Upregulation of CD38 in MM cells	Peng et al. (2023)
	BCMA-CART	ATRA leads to enhanced cytolysis, cytokine secretion and proliferation of BCMA-CAR T cells *in vitro*	ATRA Treatment Enhances BCMA Expression in MM Cells	García-Guerrero et al. (2023)
	carfilzomib	Enhancement of MM sensitivity to carfilzomib induced cytotoxicity and resensitization of carfilzomib resistant MM cells to carfilzomib *in vitro*	ATRA activates the RARγ and interferon-β response pathways, leading to upregulation of IRF1 expression	Wang et al. (2022d)
	Daratumumab	DDaratumumab-refractory MM receiving Daratumumab + ATRA had a 5% overall response rate and 66% disease stabilization	ATRA temporarily increased CD38 expression in immune cell subsets	Frerichs et al. (2021)

## 6 Ongoing clinical trials

All drugs and therapeutic regimens require clinical trials to evaluate their safety and efficacy before they can be applied to the clinic on a large scale. Previously, we have presented the results of some clinical trials of ATRA in MDS, APL, ITP and MM, some clinical trials showed that ATRA has a good prospect of application, and some clinical trials showed that ATRA does not have better efficacy. Next we summarize the ongoing clinical trials of ATRA in hematologic disorders (Table 2).

**TABLE 2:** Ongoing clinical trials of ATRA in hematologic disorders (from www.clinicaltrials.gov, Data obtained on 10 March 2024).

Compound	Sponsor	Phase	Diseases	Enrollment	Identifier
ATRA	Peking University People’s Hospital	Phase 2	ITP	80	NCT04618328
Azacitidine, ATRA	The First Affiliated Hospital of Soochow University	Phase 2	Newly diagnosed unfit AML or Intermediate, High or Very High Risk MDS	180	NCT05175508
		Phase 3			
ATRA, eltrombopag	Peking University People’s Hospital	Phase 3	Steroid-resistant/relapsed ITP	96	NCT05438875
Low-dose ATRA, attenuated dose ATO	Hospital Universitario Dr. Jose E. Gonzalez	Phase 1	APL	15	NCT05497310
		Phase 2			
TCP, ATRA, cytarabine	Michael Luebbert	Phase 1	Non-APL AML	60	NCT02717884
		Phase 2			
ATRA, ATO, Mylotarg	Associazione Italiana Ematologia Oncologia Pediatrica	Phase 2	Children and Adolescents With APL	89	NCT04793919
ATRA, ATO, Cytarabine	Institute of Hematology & Blood Diseases Hospital, China	Phase 1	Nucleophosmin-1 Mutated AML	80	NCT03031249
		Phase 2			
ATRA, ATO, Realgar-Indigo naturalis formula	First Affiliated Hospital Xi’an Jiaotong University	Phase 1	Newly diagnosed or relapsed AML	30	NCT05297123
ATRA, KPD Regimen	The First Affiliated Hospital of Xiamen University	Phase 2	Refractory/Relapsed (R/R) MM	25	NCT06158412
Realgar-Indigo naturalis formula, ATRA, ATO, Hydroxyurea	First Affiliated Hospital Xi’an Jiaotong University	Phase 3	Non-high-risk APL	110	NCT02899169
ATRA, realgar-indigo naturalis formula, etoposide or daunorubicin	Peking University People’s Hospital	Not Applicable	Low-risk APL	74	NCT05832320
Laboratory Biomarker Analysis, ATO, ATRA, Gemtuzumab Ozogamicin	M.D. Anderson Cancer Center	Phase 2	APL	151	NCT01409161
Sirolimus, ATRA	Peking Union Medical College Hospital	Phase 2	Auto-Immune Anemia	50	NCT04324411
		Phase 3			
TAS1440, ATRA	Astex Pharmaceuticals, Inc	Phase 1	R/R AML	80	NCT04282668
Chidamide, venetoclax	Shanghai Jiao Tong University School of Medicine	Phase 2	ATRA and ATO Resistant APL	30	NCT05881265
Roxadustat, ATRA	Peking Union Medical College Hospital	Phase 1	Refractory Low-risk MDS	25	NCT06020833
		Phase 2			
Compound Realgar-Indigo Naturalis Formula, ATRA	Peking University People’s Hospital	Phase 3	APL	109	NCT04175587
Decitabine, Cytarabine, ATRA, G-CSF	Shanghai Tong Ren Hospital	Phase 2	AML and MDS with blast excess	50	NCT03356080

We find that there are still many ongoing clinical studies of ATRA, including lung cancer, pancreatic cancer, skin diseases, and more. In the hematologic system, there are 7 in APL, 4 in non-APL AML, 3 in MDS, 2 in ITP, 1 in auto-immune anemia, and 1 in MM. All of these studies were combined drug treatments.

## 7 Conclusion

ATRA is an ancient and miraculous drug, which was first used for the treatment of a number of skin diseases, and later applied in the treatment of tumors, central nervous system diseases, metabolic diseases and so on. ATRA acts by inducing autophagy signaling pathway, angiogenesis, cell differentiation, apoptosis, and immune function. In the hematologic system, ATRA was the first cell differentiation drug approved for the treatment of APL, and ATRA has worked wonders to transform APL from a disease with a very high mortality rate to the most efficacious AML. With various studies, ATRA has been gradually applied in the treatment of ITP, AA, MDS, MM, lymphatic system diseases, and non-APL AML. Particularly in ITP, as a disease prone to recurrent relapses, the addition of ATRA can significantly improve initial and long-term responses in relapse-refractory patients and can reduce relapse rates. In MM, MDS and non-APL AML, the effect of ATRA drug therapy seems to be less obvious, but recent studies have found that ATRA can increase the expression of CD38 and BCMA in tumor cells, which improves the efficacy of daratumumab, CD38-CART, and BCMA-CART, which is encouraging news. There are still many ongoing clinical trials of ATRA in hematologic diseases, and it is believed that ATRA will be more widely used in hematologic diseases in the future.
